# Factors associated with the speed and scope of diffusion of COVID-19 therapeutics in a nationwide healthcare setting: a mixed-methods investigation

**DOI:** 10.1186/s12961-022-00935-x

**Published:** 2022-12-14

**Authors:** Jennifer La, Nathanael R. Fillmore, Nhan V. Do, Mary Brophy, Paul A. Monach, Westyn Branch-Elliman

**Affiliations:** 1 VA Boston Cooperative Studies Program, Boston, MA United States of America; 2 Department of Medicine, VA Boston Healthcare System, 1400 VFW Parkway, West Roxbury, Boston, MA 02132 United States of America; 3 Dana Farber Cancer Institute, Boston, MA United States of America; 4 Harvard Medical School, Boston, MA United States of America; 5 Boston University School of Medicine, Boston, MA United States of America; 6 VA Boston Center for Healthcare Organization and Implementation Research, Boston, MA United States of America

**Keywords:** Diffusion, Dissemination, COVID-19, Therapeutics

## Abstract

**Background:**

The global COVID-19 pandemic is an opportunity to evaluate factors associated with high levels of adoption of different therapeutics in a real-world setting. The aim of this nationwide, retrospective cohort study was to evaluate the diffusion and adoption of novel therapeutics with an emerging evidence basis and to identify factors that influenced physicians’ treatment decisions.

**Methods:**

*Cohort creation*: A cohort of Veteran patients with a microbiologically confirmed diagnosis of SARS-CoV2 were identified, and cases were classified by disease severity (outpatient, inpatient with mild and severe disease, intensive care unit ICU]). After classification of disease severity, the proportion of cases (outpatients) and admissions (inpatients) in each category receiving each type of medication were plotted as a function of time. *Identification of milestones and guidance changes*: Key medications used for the management of COVID-19 milestones in the release of primary research results in various forms (e.g. via press release, preprint or publication in a traditional medical journal), policy events and dates of key guidelines were identified and plotted as a timeline. After a timeline was created, time points were compared to changes in medication use, and factors potentially impacting the magnitude (i.e. proportion of patients who received the treatment) and the speed (i.e. the slope of the change in use) of practice changes were evaluated.

**Results:**

Dexamethasone and remdesivir, the first two medications with clinical trial data to support their use, underwent the most rapid, complete and sustained diffusion and adoption; the majority of practice changes occurred after press releases and preprints were available and prior to guideline changes, although some additional uptake occurred following guideline updates. Medications that were not “first in class”, that were identified later in the pandemic, and that had higher perceived risk had slower and less complete uptake regardless of the strength and quality of the evidence supporting the intervention.

**Conclusions:**

Our findings suggest that traditional and social media platforms and preprint releases were major catalysts of practice change, particularly prior to the identification of effective treatments. The “first available treatment in class” impact appeared to be the single most important factor determining the speed and scope of diffusion.

**Supplementary Information:**

The online version contains supplementary material available at 10.1186/s12961-022-00935-x.

## Contributions to the literature

- Limited empirical data are available regarding factors associated with rapid dissemination and diffusion in healthcare.
- Adoption of first-in-class therapeutics occurred rapidly and was sustained, after press releases, coverage by traditional and social media outlets, and preprints, and prior to peer-reviewed publication and guideline updates.
- High-quality evidence generated later had a slower rise and lower peak uptake, indicating practice change is harder after a clinical niche has been filled.
- The Speed and Scope of Diffusion Matrix created and presented could be applied in other settings to predict the speed and spread of novel therapeutics.

## Background

SARS-CoV-2 was first identified as a coronavirus on 12 January 2020, and WHO declared a global pandemic on 11 March 2020 [1]. At the time of the declaration, no treatments supported by high-quality evidence were available to manage the novel infection. However, according to the COVID-19 WHO database [2], during the approximately 2.5 years since the pathogen was first identified and characterized, more than 6600 clinical studies have been conducted or are underway in a race to identify effective interventions [2].

There is limited empirical evidence to explain how and why different clinical interventions were translated rapidly into patient care and why other interventions were not used, or uptake was more limited; however, several frameworks and theories suggest factors that may influence speed and scope of diffusion of innovations. The Designing for Accelerated Translation (DART) [3] of emerging innovations in health framework aims to improve upon the well-described 17-year lag between evidence generation and implementation into practice [4], and highlights several factors that theoretically impact pace of dissemination, including demand, risk, cost and the evolving evidence base. The DART framework suggests that the pace of translation of evidence into practice is a function of the strength of the underlying evidence about the effectiveness of the intervention, the demand for the intervention (including the urgency of the need and availability of alternative options) divided by the sum of risks and costs of the intervention. The diffusion of innovations theory [5] applied to medical practice describes factors associated with passive uptake of novel evidence into clinical care and highlights the role of early adopters and influencers on advancing uptake of new ideas. Greenhalgh et al.'s *Diffusion of Innovations in Service Organizations* [6] identifies various features of the innovation that impact uptake, including level of evidence, relative advantage compared to existing treatments, compatibility with clinical needs, knowledge required to use the intervention and complexity of administration and monitoring, among others.

The global pandemic—with unprecedented speed of evidence generation, sharing, dissemination and uptake—is an opportunity to empirically evaluate different factors associated with high levels of adoption or de-adoption of novel therapeutics and clinical evidence in a real-world setting. Thus, the aim of this national retrospective, mixed-methods study conducted within the national Department of Veterans Affairs (VA) healthcare system was to evaluate the uptake and spread of novel therapeutics with an emerging evidence basis released and presented via a variety of mechanisms, and to identify factors that influenced the speed and scope of practice change. A secondary goal was to use the quantitative data to inform the development of a factor scoring matrix using themes identified in prior studies evaluating factors influencing diffusion that can be used to assess the likely scope and spread of an intervention based on its inherent characteristics and various contextual factors that impact clinical practice change.

## Methods

### Overview

The aim of this national, retrospective mixed-methods study was to assess the impact of different factors with a theoretical basis for impacting clinical practice patterns, including availability, quality and strength of evidence, perceived clinical needs, and guideline endorsements on the speed and scope of the diffusion of COVID-19 treatments. First, based on a review of national guidelines and input from physician collaborators (WBE and PM), key medications used for the management of COVID-19 were identified, focusing on medications used to treat inpatients with severe disease. After a list of medications was generated, key milestones in the release of primary research results in various forms (e.g. via press release, preprint or publication in a traditional medical journal), policy events (date of United States Food and Drug Administration [FDA] Emergency Use Authorization [EUA] when applicable) and dates of key guidelines for the management of inpatient COVID-19 published by the United States National Institutes of Health (NIH) were identified and plotted longitudinally over time. Release dates were identified via internet searches, searches of social media postings (i.e., Twitter), news reports and releases, searches of preprint servers and searches on journal websites. After creation of a timeline, key milestones were compared to changes in the proportion of medication administrations within the national VA healthcare system. Factors potentially impacting the magnitude (i.e. proportion of admissions who received the treatment) and the speed (i.e. the slope of the change in use) of practice changes were subsequently evaluated using an iteratively adapted diffusion of innovations matrix, which was developed using factors included in established frameworks and theories.

### Summary of COVID-19 therapeutics

COVID-19 therapeutics fall into several broad categories: antivirals (e.g. remdesivir), anti-inflammatory medications (e.g. dexamethasone, tocilizumab, baricitinib), immunological therapies (e.g. monoclonal antibodies) and medications to prevent the sequelae of COVID-19 (e.g. anticoagulants, such as heparin). Early in vitro data suggested that hydroxychloroquine and ivermectin might also have antiviral properties [7, 8]. Based on the kinetics of the infection and the resulting clinical syndrome, as well as knowledge from the management of other infectious diseases, it was postulated very early after the identification of SARS-CoV-2 that antivirals were likely to be most effective when administered early in the disease course and anti-inflammatory medications likely to be more effective during later stages of the illness; the expected mechanisms underlying disease progression impacted which medications were indicated in which patient populations and when administration was appropriate (e.g. antivirals recommended only for use early in the treatment course). Due to coding challenges with administration and rapid changes related to multiple products and evolution of variants, monoclonal antibodies and convalescent plasma were not assessed. Additionally, other medications associated with management of complications, such as heparin prophylaxis, were not evaluated, as these are routinely given to hospitalized patients, and changes would be difficult to identify and attribute to any particular change in evidence or policy.

### Coding of clinical guidelines

Throughout the pandemic, the national VA healthcare system recommended application of the NIH inpatient treatment guidelines [9] to direct management of patients with COVID-19; of note, early in the pandemic, and prior to the identification of any evidence-based treatments, many facilities developed local processes and internal treatment guidance [10]. Two physicians (WBE and PM) developed a coding scheme for classifying the NIH treatment guidelines (see Additional file 1, Materials for example data entry form). Data extracted included date of recommendation, medication, strength of recommendation based on standard grading schemes applied by the NIH, quality of recommendation as assessed by the NIH, population included in the recommendation (e.g. disease severity) and additional caveats and notes.

After qualitative coding of the NIH guidelines, antiviral and anti-inflammatory medications with strong recommendations for and against their use in guidelines and those with a priori interest based on widespread discussion, such as ivermectin and hydroxychloroquine, were evaluated for uptake quantitatively using VA medication administration data.

### Cohort creation

All Veteran patients with a documented SARS-CoV-2-positive clinical test during the period from 1 March 2020 to 1 May 2022 were identified using the national VA COVID-19 Shared Data resource (see STROBE [STrengthening the Reporting of OBservational studies in Epidemiology] checklist, Additional file 2) [11]. Because recommendations for medication administration vary depending upon disease severity, patients with a COVID-19-positive microbiological test were then classified based on disease severity: intensive care unit (ICU) patients with severe disease, other inpatients with and without evidence of severe disease, and outpatients. Inpatient disease severity was evaluated using previously described methods based on oxygen saturation (SpO_2_) levels and receipt of supplemental oxygen, similar to NIH disease severity designations [12]. Briefly, inpatient management was defined as admission to a VA acute-care hospital within 14 days of a positive test or any positive test during an inpatient admission. Outpatient management for the purposes of measuring medication administration was defined as any positive COVID-19 microbiological test that did not meet the definition of an inpatient COVID-19 hospitalization. Severe inpatient disease was defined by documented SpO_2_ < 94% or receipt of any supplemental oxygen during a window of –1 to 14 days after any positive test. Any patient admitted to the ICU during the admission was classified as “ICU.” Inpatients not meeting criteria for severe disease were classified as having non-severe COVID-19. Given concerns about missing vital signs data leading to misclassification, patients admitted to the ICU but without evidence of respiratory compromise were excluded.

Medication administrations (inpatients) and dispenses (outpatients) were extracted from the VA Corporate Data Warehouse, the VA’s national research data repository. Proportions of patients (for outpatients) or admissions (for inpatients) in each disease severity group who received the medication were plotted over time by calendar week. For the medications where changes occurred particularly rapidly, administrations were plotted on a daily basis in order to demonstrate the exact times that practice patterns began to change so that the impact of different types of information releases and sharing mechanisms (e.g. press release/preprint release versus official publication) could be evaluated. No attempt was made to identify whether patients received multiple medications simultaneously, since the goal of the study was to evaluate speed and scope of practice change as a function of time and key timeline milestones rather than compliance with a specific set of clinical guidelines.

### Quantitative analysis

For key timeline events, changes in practice patterns were evaluated relative to the timing of different events (e.g. press release, preprint release, publication, guideline change) to determine which factors were most strongly impacting clinical practices. Of note, releases on social media, via preprint servers and via press releases tended to occur nearly simultaneously, while traditional peer-reviewed publication and guideline updates were relatively delayed.

Visually assessed changes in the slope of the proportion of patients receiving a medication were used to assess the speed of uptake. Scope of uptake was evaluated using the proportion of patients who received the medication, irrespective of the initial slope of practice change. Impacts of information availability (e.g. from preprints/social media, traditional peer-reviewed publication, guideline updates) were evaluated by correlating the event to changes in the proportion of patients receiving a medication. Chi-squared tests with of proportions with continuity corrections were used to evaluate differences in uptake. Quantitative analysis was completed using R version 4.1.2.

### Adapted Speed and Scope of Diffusion Matrix

An adapted Speed and Scope of Diffusion Matrix was created using the Greehalgh et al. systematic review of factors influencing diffusion of innovations in service organizations and elements from Dubois’ framework for understanding the pace of adoption and was then iteratively developed and factors graded [6, 13]. Factors from Greenhalgh et al. included relative advantage of the innovation compared to existing treatments, perceived compatibility with clinical needs, observability of impact of intervention, knowledge required to use the intervention, and intervention complexity. Definitions of each of the variables are included in Additional file 4: Table S1. Factors identified in the Greenhalgh et al. systematic review but found to be not relevant to one-time medication administrations, such as those related to complex interventions and long-term sustainability, were not evaluated or coded. In alignment with concepts presented in the DART framework, perceived risk of the intervention, level of evidence, strength of guideline recommendation, and presence of conflicting evidence were also included. In addition to factors identified from these existing theories and frameworks, strength and quality of the evidence as assessed by the NIH Treatment Guidelines committee and key factors felt by clinicians (WBE and PM) to be potentially associated with diffusion, such as the biological basis and rationale for the treatment and a conflicting evidence base, were included. If the Speed and Scope of Diffusion Matrix score did not explain the data, the matrix was iteratively adapted (e.g. additional factors added) until the matrix rankings (e.g. most to least likely to be adopted) were reflective of the real-world data. Impacts of release of different types of information release (e.g. via preprints versus traditional peer-reviewed publication) were only assessed in the quantitative aspects of the analysis.

After key factors influencing diffusion were identified, a matrix was created which rated each of the included factors as strongly supportive, supportive, neutral, against and strongly against. Scores were primarily assigned by one study author (WBE) with review by a second author (PM). Disagreements were resolved through internal discussion. The scored system was then compared to the speed and scope of the diffusion of medications (quantitative data, slope of the curve during a clear period of change in use) as an internal verification.

### Ethical considerations

The study was approved by the VA Boston Institutional Review Board (IRB) as an exempt human research study (IRB #3328-X) prior to data collection and analysis.

## Results

### Quantitative findings

**Fig. 1:**
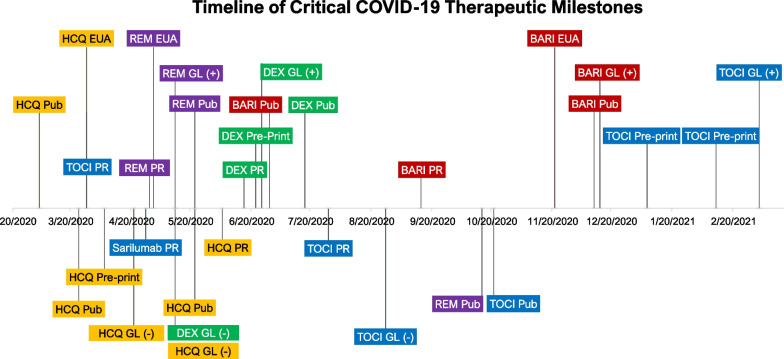
A timeline of critical COVID-19 therapeutic milestones. The *x*-axis is presented longitudinally. Factors that supported uptake are presented above the *x*-axis, and factors that supported lack of uptake or de-adoption are presented below the *x*-axis. Different treatments are represented by different colours (yellow: HCQ, hydroxychloroquine; blue: TOCI, tocilizumab; purple: REM, remdesivir; green: DEX, dexamethasone; red: BARI, baricitinib). The height of the different timeline elements above or below the axis represents the information or guideline type; press releases (PR) are the closest to the *x*-axis, followed by preprints, peer-reviewed publications (Pub), guideline changes (GL) and EUAs issued by the FDA. Due to horizontal space limitations, some key milestones in the identification of baricitinib as an effective treatment are not shown (Marconi et al. COV-BARRIER preprint [3 May 2021] and publication [1 September 2021]). ACTT-4, which showed equivalence of baricitinib vs dexamethasone with fewer adverse events among patients receiving baricitinib, was published on 22 May 2022

**Fig. 2:**
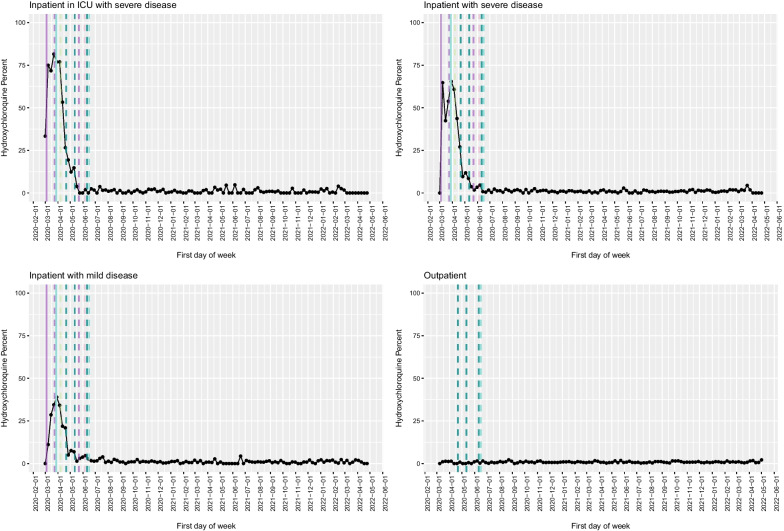
Hydroxychloroquine use in the VA as a function of time, with key milestones noted. Proportion of COVID-19 cases that received hydroxychloroquine administration by week among inpatients in the ICU (**A**), inpatients with severe disease (**B**), inpatients with mild disease (**C**) and outpatients (**D**). Solid lines represent factors that supported adoption, and dashed lines represent factors that favoured de-adoption. Different colours represent different types of information sources (light blue: FDA EUA; dark blue: NIH guideline recommendation; dark green: preprint posting date; purple: publication in peer-reviewed journal; pink: press release)

**Fig. 3:**
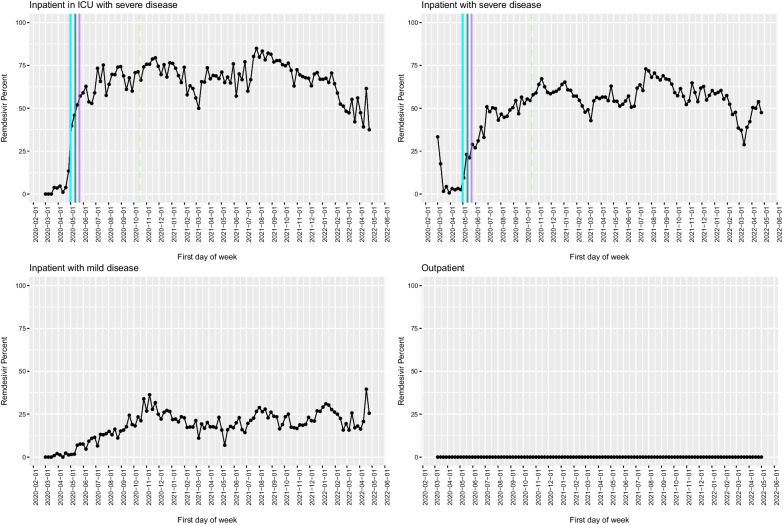
Remdesivir use in the VA as a function of time, with key milestones noted. Proportion of COVID-19 cases that received remdesivir by week among inpatients in the ICU (**A**), inpatients with severe disease (*B*), inpatients with mild disease (**C**) and outpatients (**D**). Solid lines represent factors that supported adoption, and dashed lines represent factors that favoured de-adoption. Different colours represent different types of information sources (light blue: FDA EUA; dark blue: NIH guideline recommendation; dark green: preprint posting date; purple: publication in peer-reviewed journal; pink: press release)

**Fig. 4:**
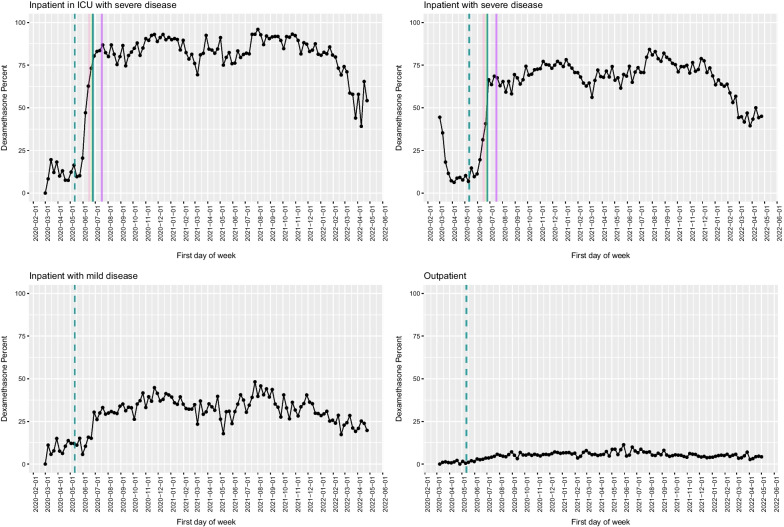
Dexamethasone use in the VA as a function of time, with key milestones noted. Proportion of COVID-19 cases that received dexamethasone by week among inpatients in the ICU (**A**), inpatients with severe disease (**B**), inpatients with mild disease (**C**) and outpatients (**D**). Solid lines represent factors that supported adoption, and dashed lines represent factors that favoured de-adoption. Different colours represent different types of information sources (light blue: FDA EUA; dark blue: NIH guideline recommendation; dark green: preprint posting date; purple: publication in peer-reviewed journal; pink: press release)

**Fig. 5:**
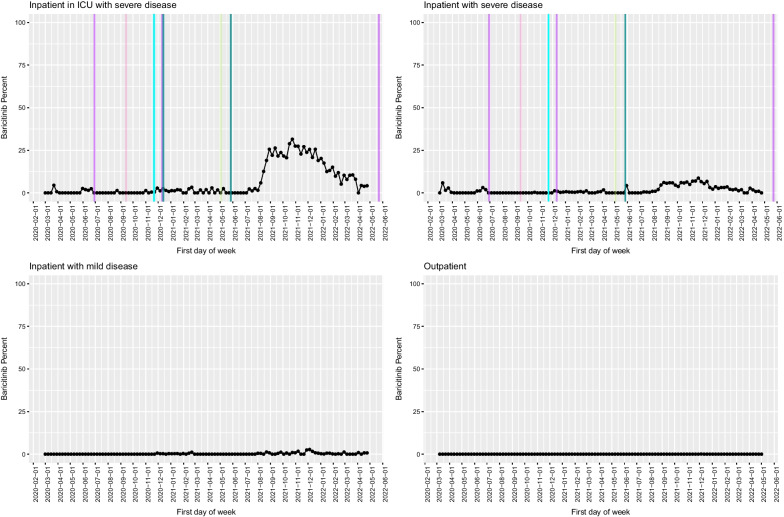
Baricitinib use in the VA as a function of time, with key milestones noted. Proportion of COVID-19 cases that received baricitinib by week among inpatients in the ICU (**A**), inpatients with severe disease (**B**), inpatients with mild disease (**C**) and outpatients (**D**). Solid lines represent factors that supported adoption, and dashed lines represent factors that favoured de-adoption. Different colours represent different types of information sources (light blue: FDA EUA; dark blue: NIH guideline recommendation; dark green: preprint posting date; purple: publication in peer-reviewed journal; pink: press release)

**Fig. 6:**
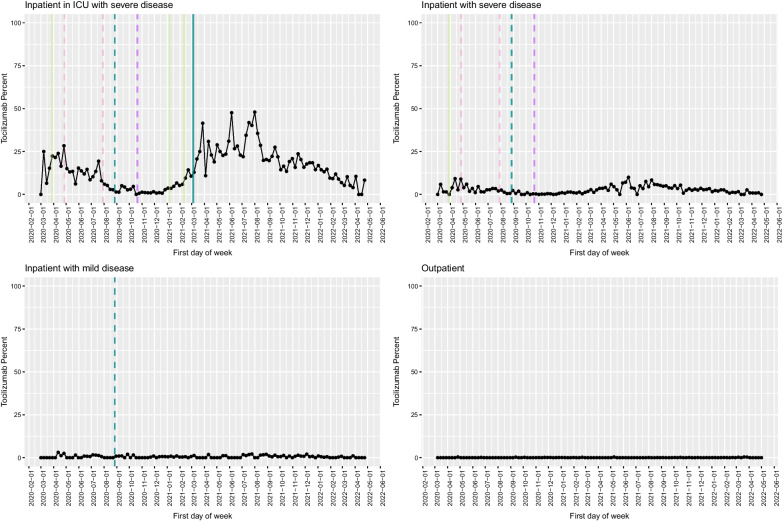
Tocilizumab use in the VA as a function of time, with key milestones noted. Proportion of COVID-19 cases that received tocilizumab by week among inpatients in the ICU (**A**), inpatients with severe disease (**B**), inpatients with mild disease (**C**) and outpatients (**D**). Solid lines represent factors that supported adoption, and dashed lines represent factors that favoured de-adoption. Different colours represent different types of information sources (light blue: FDA EUA; dark blue: NIH guideline recommendation; dark green: preprint posting date; purple: publication in peer-reviewed journal; pink: press release)

During the study period, 169,304 Veterans had 191,625 positive COVID-19 tests. Among these, 103,529 were outpatients without documentation of severe disease. There were 21,126 inpatient admissions without evidence of respiratory compromise (non-severe), 36,040 inpatient admissions with severe disease but not requiring ICU admission, and 13,254 ICU admissions. A timeline of potentially pivotal COVID-19 therapeutics milestones is presented in Table 1 and plotted in Fig. 1; the proportions of patients with different levels of disease severity who received the medications of interest (hydroxychloroquine, remdesivir, dexamethasone, baricitinib, tocilizumab) are presented in Figs. 2, 3, 4, 5 and 6. Ivermectin use was rare, and it was not further evaluated (Additional file 5: Figure S1).

**Table 1:** Timeline of key COVID-19 treatment guidelines

Milestone date	Medication	Type
4 March 2020	Hydroxychloroquine publication: suggesting theoretical benefit	Publication [7]
24 March 2020	Hydroxychloroquine publication: questioning potential for harm	Publication [40]
28 March 2020	Hydroxychloroquine: FDA EUA	EUA [43]
28 March 2020	Tocilizumab: publication of case series	Preprint/Twitter [44]
16 April 2020	Chloroquine: preprint of clinical data suggesting harm	Preprint [14]
21 April 2020	Hydroxychloroquine: NIH treatment guidelines recommend against use in combination with azithromycin	Guidelines [45]
27 April 2020	Sarilumab: press release dropping low-dose arm due to potential harm	Press release [25]
29 April 2020	Remdesivir: press release of ACCT-1 study results	Press release [46]
1 May 2020	Remdesivir: FDA EUA	EUA [19, 37]
12 May 2020	Dexamethasone: NIH treatment guidelines recommend against use of corticosteroids except in acute respiratory distress syndrome	Guidelines
12 May 2020	Hydroxychloroquine: NIH treatment guidelines recommend against use	Guidelines
22 May 2020	Hydroxychloroquine: publication of Surgisphere data in *Lancet* (later retracted)	Publication [15, 16]
12 May 2020	Remdesivir: NIH treatment guidelines recommend use in patients with severe disease	Guidelines
22 May 2020	Remdesivir: publication in *New England Journal of Medicine*	Publication [17]
5 June 2020	Hydroxychloroquine: press release stating initial results from RECOVERY trial failed to demonstrate a benefit	Press release [47]
16 June 2020	Dexamethasone: press release stating preliminary results of recovery trial demonstrate benefit	Press release [48]
22 June 2020	Dexamethasone: RECOVERY preprint posted	Preprint [18]
25 June 2020	Dexamethasone: NIH treatment guidelines recommend use in severe disease	Guidelines
29 June 2020	Baricitinib: pilot study published in *Clinical Infectious Diseases* demonstrating possible benefit	Publication [31]
17 July 2020	Dexamethasone: Publication of RECOVERY Results in the *New England Journal of Medicine*	Publication [49]
29 July 2020	Tocilizumab: results of COVACTA released with no evidence of benefit	Press release [24]
27 August 2020	Tocilizumab: NIH treatment guidelines recommend against use	Guidelines
14 September 2020	Baricitinib: early results of trial released via press release demonstrating modest reduction in hospital length of stay	Press release [29]
8 October 2020	Hydroxychloroquine: results of RECOVERY trial published demonstrating no benefit	Publication [50]
15 October 2020	Remdesivir: results of WHO study released demonstrating no benefit	Preprint [51]
21 October 2020	Tocilizumab: results of clinical trial published demonstrating no benefit	Publication [23, 52]
19 November 2020	Baricitinib: FDA EUA	EUA [53]
11 December 2020	Baricitinib: ACCT-2 study results published in *New England Journal of Medicine*	Publication [28]
14 December 2020	Baricitinib: NIH treatment guidelines recommends limited use in limited populations	Guidelines
7 January 2021	Tocilizumab: REMAP-CAP trial preprint demonstrating a benefit in combination with dexamethasone	Preprint [26]
11 February 2021	Tocilizumab: RECOVERY preprint posted demonstrating a benefit in combination with dexamethasone	Preprint [27]
5 March 2021	Tocilizumab: NIH treatment guidelines recommend for use in ICU patients	Guidelines
3 May 2021	Baricitinib: COV-BARRIER preprint posted demonstrating a benefit in combination with dexamethasone	Preprint [30]
27 May 2021	Baricitinib: NIH treatment guidelines recommend use for patients with severe disease in combination with dexamethasone	Guidelines
23 May 2022	Baricitinib: publication in *Lancet Respiratory Medicine* demonstrating a benefit of baricitinib over dexamethasone	Publication [32]

Hydroxychloroquine use was high during March of 2020, with both a steep rate of rise and a steep rate of decline; the rapid de-adoption of use for inpatients with COVID-19 appeared to occur immediately following the release of a preprint that suggested a potential for worse outcomes in patients who received high-dose chloroquine in combination with azithromycin [14]; by the time initial clinical trials data demonstrating lack of effectiveness were available and NIH treatment guidelines were updated in the middle of May 2020, administrations had fallen from a peak of 81.5% of ICU admissions during the last week of March 2020 to 3.9%. During the same time period, similar declines were seen among lower-acuity admissions and outpatients (inpatient, severe, from 53.9 to 3.7%; inpatient, non-severe, from 34.5 to 1.4%; outpatients from 1.32 to 0.52%). Notably, by the time the widely publicized Surgisphere study [15, 16]—which raised concern for harm with any dose of hydroxychloroquine before being retracted—was available, use was already quite rare in the VA, and thus the impacts of this high-profile study on clinical practice patterns were minimal.

**Fig. 7:**
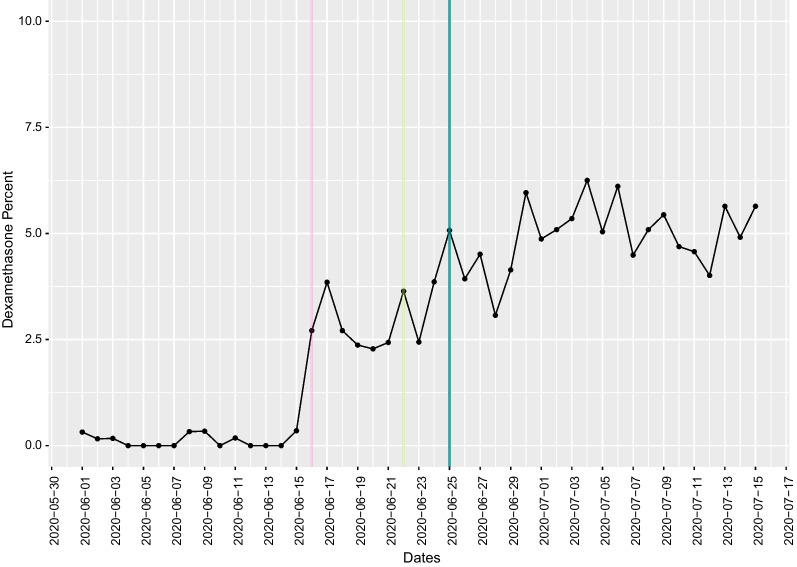
Dexamethasone new administrations per day during the period from 1 June 2020 to 17 July 2020. Proportion of all inpatients that received dexamethasone by day during the period from 1/6/2020 to 17/7/2020. The pink line represents the initial press release stating the treatment was beneficial, the light green line represents the day the preprint was posted, and the dark green line represents the day the NIH guidelines were updated to recommend dexamethasone to inpatients with severe disease

The first treatment with data from a randomized controlled clinical trial to support its use was the antiviral remdesivir [17], followed closely by the glucocorticoid dexamethasone (which was already FDA-approved for other uses) [18]; among medications with clinical trial data to support use, the slopes of uptake (indicating speed) and the proportion of patients who received either of these medications (indicating scope) were the steepest. Dexamethasone use rapidly increased to 0.35% of all admissions (including COVID and non-COVID admissions) prior to a press release announcing early trial results, to 2.7% on 16 June 2020, the day of its announcement in the United Kingdom, and then stabilized at approximately 2.4% of all inpatient admissions 1 week later, and use was sustained over time (Figs. 4 and 7). Increased use occurred rapidly after a press release and preprint were available, with an additional less steep rise in slope following the guideline update. No impact of the peer-reviewed publication, which was the latest milestone to be achieved, on changes in slope was identified. Administrations of remdesivir, which was investigational and therefore not available outside of a research study even with evidence supporting its effectiveness available, rose rapidly following the FDA EUA (Fig. 3) [19]. The delay in administration following the release of preliminary clinical trial results is likely due to the lack of availability before the FDA approved its use. Of note, there was no apparent decline in use of remdesivir after a VA study suggested no benefit [20], and the WHO randomized controlled trial also found no benefit [21].

As noted in the timeline and Fig. 1, the IL-6R-inhibitor, tocilizumab, had conflicting clinical trial results and variable guideline support over time. Initially, the medication was recommended for use based on early reports from China and its effectiveness for reducing mortality in other hyper-inflammatory states [22, 23], but after two discouraging press releases from manufacturers regarding their own trials [24, 25], use fell substantially until two additional clinical trials were published suggesting a benefit in combination with treatments above that had become standard of care [26, 27]. The maximum proportions of admissions with severe disease (10.0%) and ICU-level care (48.0%) were substantially lower than those achieved with dexamethasone and remdesivir (*P* < 0.001 for all four comparisons), and the slope of adoption was substantially less steep.

All clinical trials of the anti-inflammatory JAK inhibitor baricitinib have consistently demonstrated clinical benefit, and very recently the medication was found to be safer than dexamethasone [28–32]. Thus, the evidence basis supporting baricitinib is the highest quality and the most extensive. However, the earliest evidence supporting its use was from a trial in which it was combined with remdesivir but not dexamethasone, and the trial was conducted substantially later in the pandemic, after clinical practice patterns were already established and perception of clinical need may have been lower [28, 29]. During the study period, baricitinib was used relatively infrequently in comparison to dexamethasone, and uptake did not increase until a trial in which it was given with dexamethasone [30] was published as a preprint and integrated into the NIH guidelines. The slope of baricitinib uptake was substantially less steep and overall use relatively low and primarily limited to ICU patients (maximum uptake for baricitinib in ICU patients, 31.5 vs 96.0% in dexamethasone, *P* < 0.001). The time to maximum uptake of dexamethasone in the ICU occurred more rapidly after press release and preprint than after similar milestones for baricitinib.

### Qualitative findings

Factors included in the adapted Speed and Scope of Diffusion Matrix are presented in Table 2. As theoretically suggested in the DART framework heuristic, speed and degree of use were positively associated with high scores with clinical evidence supporting use in the absence of other treatments in the class, biological and clinical plausibility, perceived compatibility with current clinical needs, and observability of improvements in clinical outcomes and inversely associated with perceived costs and risks. Although not specifically included in the diffusion matrix, but related to perceived clinical need and urgency, underlying disease severity of the patient also appeared to play a role; for example, administration of hydroxychloroquine was highest among ICU patients and lowest among outpatients with a clear trend toward more use in higher-acuity patients. Factors associated with slower and less substantial uptake were conflicting evidence, predicted lower familiarity with the drug or similar drugs, and perceived risk associated with the medication, also supporting the role of perceived risks and costs as factors that slow the speed and scope of uptake. The total positive versus negative ratings in the framework appeared to be positively associated with the speed and degree of medication adoption.

**Table 2:**
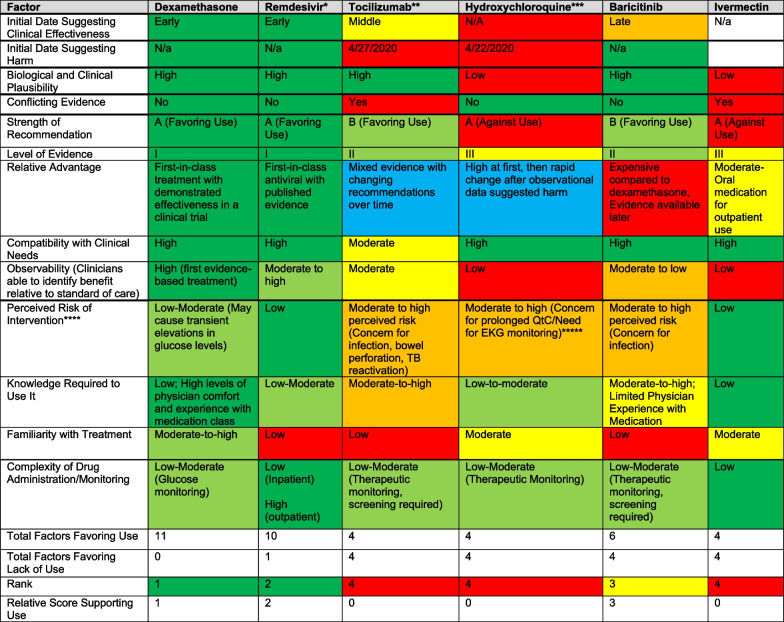
Scope and Speed of Diffusion Matrix

Colour coding scheme as follows: dark green—supports uptake of the intervention; light green—supports uptake of intervention, but less strongly; yellow—neutral impact on uptake; orange—weak factor against uptake; red—strong factors against uptake; blue—mixed impacts and impacts that may have changed over time

*Initial strength of evidence grade was III; however, lower strength of evidence grade was found prior to FDA approval, and thus experimental medication was not widely available, as the compassionate use program was discontinued very early on

**Quality and strength of evidence rankings were variable for tocilizumab, depending upon the data available at the time and the population being studied

***Strength of evidence and quality of evidence rankings were the same before and after the recommendation against use

****Perceived clinical risk; actual risk is likely similar to tocilizumab and baricitinib, but clinical providers are generally more familiar with corticosteroids than the other anti-inflammatory medications, which tend to be prescribed and managed primarily by specialists

******Reflects risk perception, and not actual risk. Note that this changed over time; initially felt to be minimal risk, but risk perception was driven by a high-profile study that demonstrated harm. Although this investigation was later retracted, the impacts were sustained

## Discussion

In the setting of a worldwide emergency, practice changes initially diffused at a rapid pace in the United States Veterans Health Administration; after effective medications in the two key different classes (anti-inflammatory and antiviral) became available, the speed and scope of diffusion and practice change slowed considerably. Although the emergency context in which this study was conducted is not typical, the initial lack of any evidence-informed treatment options offers an opportunity to study factors that may impact practice change that are not possible to evaluate for conditions with long treatment histories. For many diseases, treatment options are available, and therefore the “first treatment” or “first in class” effects cannot be studied. The global pandemic offers a rare opportunity to study factors that impact practice change, especially the role of the “relative advantage” of filling a perceived clinical niche. We found that these impacts on both speed and scope of uptake were substantial and durable. Practice patterns were also likely influenced by internal factors (e.g. local opinion leaders and treatment algorithms/order sets) and external factors (e.g. information ecosystem, political pressure, and context), although these were not specifically assessed in this study. The empirical results presented in this study also broadly support elements of the heuristic proposed in the DART framework, which highlights the interactions between evidence base, demand for innovation, and risks and costs. The Speed and Scope of Diffusion Matrix provides a means for ranking various factors to predict the rate and degree of dissemination of innovations in healthcare.

In the FAST framework for considering factors that drive the speed of practice change, Proctor et al. highlight different systems and contextual factors that theoretically impact speed and scope of information sharing. The framework highlights factors associated with accelerated diffusion, including clinical demand, evidence strength, clinical need and urgency and also with decelerated change, including harms, costs and provider risk aversion [33]. Our study adds to the current literature by including real-world data about the adoption and de-adoption of various therapeutic options and correlating changes with specific events and internal and external factors that were associated with these changes to provide empirical, real-world evidence for these theoretical frameworks. The explanatory Speed and Scope of Diffusion Matrix tool could be used and refined in future studies to predict the how practice change may occur when new medications and interventions become available based on characteristics of the intervention and the context in which it is being introduced.

**Fig. 8:**
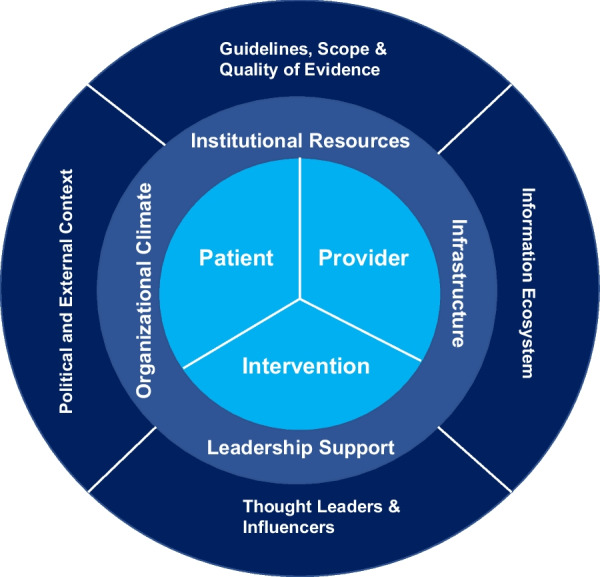
Theoretical model of factors impacting the speed and scope of diffusion

While we conducted a granular analysis of changes in practice patterns over time within the national VA healthcare system and correlated these changes with factors at the healthcare facility level and key milestones, we were not able to assess many of the factors known to impact clinical practice patterns, including internal organizational factors, the role of key opinion leaders, and internal facility guideline teams. In addition, we were not able to capture external political factors which may also have impacted clinical decision-making (see Fig. 8 for a theoretical model of internal and external factors influencing diffusion).

Dubois et al. highlighted the role of science communication and dissemination of new evidence through key thought leaders as major drivers of adoption and de-adoption [13]. In part due to the speed at which information from clinical trials was disseminated, the subsequent changes in guidelines and peer-reviewed publication in traditional medical journals appeared to have a modest or negligible impact on adoption when compared to other information sources, including press releases, posting to preprint servers and sharing of information via social media platforms. For example, administration of dexamethasone began to increase on the same day as the press release and reached close-to-peak use the following day. Use was mostly sustained at high levels, with only a small increase in uptake occurring following the guideline updates (Fig. 7). We are not able to quantify the separate impacts of the preprints, social media and traditional media sources in the information ecosystem, as these interacted, and information availability on each of these sources occurred nearly simultaneously. However, the timing of practice changes—before peer-reviewed publication and prior to integration into clinical guidelines—highlights the importance of media influences and suggests that factors that have traditionally been felt to drive clinical practice change, such as the quality of the evidence and high-profile peer-reviewed publication, may have less substantial impact than in the past, when information was shared in different ways. It is unclear if the strong influence of social and traditional media coverage found in this study will generalize to nonemergency settings without the same degree of media attention and social medial sharing, but physician practice may continue to be impacted by these networks, and the influence of these factors should be considered in future studies that aim to reduce the time from evidence generation to translation into clinical practice. We anticipate that preprints may continue to be a mechanism for sharing data and thus may have long-term impacts on how and when practice changes occur. Opinion leaders are also likely to continue to share results on social media at the preprint stage for particularly important, practice-changing studies, thus key opinion leaders and social media are likely to play an increasingly important role in influencing clinical practice in years to come. Our findings suggest that these impacts may be the most substantial for situations with high perceived clinical need and may be less substantial once practice patterns are established and perceived need and interest wanes. Our findings also have implications for de-adoption of interventions that were initially presented as effective but with a more conflicting or less convincing evidence basis over time, as we found that evidence generated later had less of an impact than evidence generated earlier and which received more attention. Additional research is needed to identify effective strategies for leveraging these influences and new information sharing patterns, particularly for changing long-established practice patterns with a changing evidence basis.

De-adoption, and incorporation of new evidence, was variable for the different medications, and likely driven by different factors, including perceived risk/benefit profile and social and traditional media influences. Hydroxychloroquine use rose quickly and was almost completely discontinued in a short time, likely due to concerns about the initial studies supporting its effectiveness, which were not based on human trials, combined with studies suggesting that the treatment might be harmful [16, 34, 35]. Its use, which was the highest among ICU patients, was likely driven in part by external influences supporting its potential benefit and in part by a bias among clinicians to “do something”, particularly among critically ill patients at high risk of death without intervention. The bias to intervene with a medication with no human data to support its use was much lower among outpatients, likely because of their overall much lower risk of severe outcomes, which created less of a sense of urgency amongst clinicians. Medication availability, familiarity with the drug and a long-standing safety track record also likely facilitated widespread uptake.

The durable impact of “relative advantage” is well demonstrated by the ongoing sustained use of the antiviral remdesivir. Remdesivir was the first antiviral to be approved under an EUA by the FDA, and later it was fully approved [17, 36, 37]. Although low-quality clinical evidence supporting remdesivir use was available before the EUA [36], it was not available for “off-label” use because it was not FDA-approved for any condition, and therefore adoption prior to approval was not possible. Once remdesivir was integrated into clinical care, it continued to be administered with approximately the same frequency, despite subsequent studies suggesting a less substantial benefit or no benefit at all [20, 38]. We are not able in this study to fully determine the reasons why subsequent high-quality evidence did not have a major impact on clinical practice, but potential explanations include ongoing strong support in the NIH treatment guidelines, continued support by local thought leaders and in locally developed and integrated processes and procedures, and anchoring effects. The fact that most major trials following the first positive trial of remdesivir were adapted to include remdesivir in all treatment groups undoubtedly influenced those providing formal or informal guidance; that is, the “anchoring” was not merely psychological. Another possibility is that as more treatments became available and outcomes improved, there was less of a sense of urgency on the part of clinicians to rapidly change practice and less focus on new studies that suggested lower effectiveness. In addition, remdesivir has a good safety record and remains the only antiviral medication approved for inpatient use, thus there is both a strong biological plausibility for its use and no competition for its clinical treatment “niche.”

The availability of therapeutic options with high-quality evidence to support their use seemed to play a major role in the slower adoption of medications for which evidence accumulated later, potentially due to several internal and external factors, corresponding to the theory that demand plays an important role in the speed and scope of adoption. The first medication to demonstrate a reduction in mortality was dexamethasone; at the time, many medical facilities had teams of local opinion leaders frequently reviewing evidence and updating internal guidance. This was coupled with high levels of coverage via traditional media sources and via social media sources (the information ecosystem) and a perception on the part of providers of a strong clinical need for any type of treatment. Subsequent to the release of the data about dexamethasone and its rapid integration into clinical practice, the anti-inflammatory medication baricitinib was found to be effective in multiple trials, and then very recently equal to dexamethasone but with fewer adverse events [32], yet despite its more robust evidence base, its adoption was slower and never reached the same peak as dexamethasone. This lower adoption may have been driven by many of the same factors that drove the rapid adoption of dexamethasone—far less perception of clinical need and availability of other treatments in the anti-inflammatory class, translating to lower demand, and less media coverage, translating to less knowledge about the intervention. These findings suggest that there is a lower barrier to initial adoption if there is a perceived treatment void and if demands are high than if a superior treatment is identified that needs to replace or even just augment an established practice. Future research is needed to identify communications strategies that can overcome the “anchoring” effect and early relative advantage.

The rapid changes in practice patterns that occurred prior to changes in national treatment guidelines and prior to publication in peer-reviewed journals suggest that factors traditionally postulated to drive practice patterns did not play a major role. Potential drivers of changes therefore include information ecosystems: traditional and social media coverage, media and local influencers, and internal algorithm development by local opinion leaders, who likely communicated with their counterparts in other hospital systems. The perceived state of emergency also played a role early on, both with regard to the rapid adoption and de-adoption of hydroxychloroquine, but also with the rapid adoption of dexamethasone and remdesivir. Delayed and less pervasive adoption of baricitinib (Fig. 8) was the one instance where use did not appear to be driven by social and traditional media influence and clearly followed a new NIH guideline and where the grading and quality of the evidence as measured in the guidance appeared to play a role. The perception that the risk of severe COVID-19 was lower and that there was a high demand for a new intervention were also both lower, likely influencing overall practice patterns.

Although not included as its own category in the Speed and Scope of Diffusion Matrix, the severity of patient disease also drove practice changes and prescribing patterns; outpatients had very limited treatment throughout the study period, potentially due in part to a lower sense of urgency on the part of treating physicians. Early in the pandemic, there were no evidence-based therapies and no vaccines. The mortality rate was correspondingly higher, and physicians may have felt more pressure to “try something” and to “act quickly” than later in the pandemic when mortality rates fell and multiple options were available.

Biological plausibility of different treatment options may also partially explain our findings, since we would expect physicians to be sceptical of surprising findings—and perhaps insufficiently sceptical of more plausible findings. COVID-19 was described early on as having an early viral replication phase followed by a later inflammatory phase. This typical clinical course of disease suggested that targeting both might improve outcomes. Further, antiviral medications, such as oseltamivir, have long been used for early treatment in influenza, and in vitro data for remdesivir suggested a strong positive effect [8]. In addition, although it was not FDA-approved, the drug had previously been tested for the management of Ebola and was demonstrated to be safe [39]. Glucocorticoids have a long history of use in critical illness, and severe COVID-19 was already known to be a highly inflammatory state, and thus providers were primed to adopt the intervention. Similarly, anti-IL-6R inhibitors such as tocilizumab have a strong track record for reducing disease severity in other inflammatory conditions [22]. JAK inhibitors like baricitinib overlap considerably with IL-6R antibodies and are one of the most wide-ranging classes of anti-inflammatory and immune-suppressive drugs, and thus there was a strong theoretical basis for its use as a sole anti-inflammatory drug—but the use of JAK inhibitors in combination with high-dose glucocorticoids is rarely indicated in other diseases, and use in combination with anti-IL-6R antibodies is unprecedented in clinical medicine. In contrast, some of the medications that were prescribed early on, such as hydroxychloroquine, had less biological plausibility; data supporting use were limited to in vitro studies and some early anecdotal reports of clinical benefit from China and Italy, which were impacted earlier than the United States. The limited biological basis was then coupled with early reports of harms [14, 40], and the combination of limited data to support effectiveness with reports of harm likely contributed to its rapid de-adoption. Similarly, the molecular basis supporting ivermectin use is limited, and clinical trial results were equivocal until a pivotal trial published in a peer-reviewed journal in 2022 reported no benefit [41, 42].

Our study is subject to several limitations. First and foremost, we did not interview prescribers to identify the reasons for their clinical decision-making. This means that we are not fully able to differentiate the role of the external information ecosystem from that of local thought leaders and embedded order sets that may have driven treatment decisions. Supply chain challenges early in the pandemic are well described; however, we were not able to assess the impact of medication availability on prescribing trends. It is possible that different patterns might have been identified, particularly for tocilizumab, if the supply chain had been more stable. Due to extremely limited prescribing within the VA, we are not able to evaluate reasons for ivermectin use (Additional file 5: Figure S1). We were also not able to assess medications that were incorporated into COVID-19 treatment guidelines but were also part of the standard of care for general medical conditions, such as deep vein thrombosis prophylaxis, which is routinely administered to inpatients. This study was conducted in the VA, which is a closed healthcare system, and processes and clinical practice patterns may differ in other settings. In addition, because this study was conducted primarily in an inpatient VA population, we were not able to assess the impact of cost on clinical treatment decisions. In other settings, particularly outpatient settings with patient co-pays, cost is likely to play an important role in behaviours. That our study evaluated pandemic treatments is both a benefit and a limitation. Findings from this study may not be generalizable to other contexts; however, the unique environment provides a natural experiment to evaluate drivers of practice change that is not possible under typical conditions. The Speed and Scope of Diffusion Matrix was assessed qualitatively, but a quantitative system was not developed. More research is needed to test and refine an evidence-based scoring system for estimating diffusion of novel interventions.

## Conclusion

Our empirical findings broadly support the theoretical heuristic presented in the DART framework, which suggests that translation of innovations in healthcare is a function of effectiveness, demand, perceived risks and costs, and also identified additional factors that impacted practice patterns. Our quantitative, real-world data about clinical practices also suggest that traditional and social media platforms, and the release of results on preprint servers, were major catalysts of practice change, particularly prior to the identification of effective treatments (dexamethasone and remdesivir), as adoption and de-adoption occurred rapidly, and often before official peer-reviewed publication or integration of a new treatment into clinical guidelines. Positive evidence released earlier appeared to have a stronger impact than evidence generated later, highlighting the importance of relative advantage as a driver of clinical practice patterns. The Speed and Scope of Diffusion Matrix can be applied in other settings to predict the speed and scope of adoption of new therapies, and to develop strategies to improve uptake, particularly for innovations that compete with an existing clinical niche.

## Supplementary Information

**Additional file 1. **COVID-19 treatment milestones.

**Additional file 2. **STROBE statement—checklist of items that should be included in reports of cohort studies.

**Additional file 3.** Full study data file.

**Additional file 4. Table S1.** Factors included in the diffusion matrix, their definitions, and impact of grading.

**Additional file 5.** Ivermectin prescribing.

## Data Availability

The limited data used to conduct these analyses are included in Additional file 3.
