# Supplementary material for: Factors associated with the speed and scope of diffusion of COVID-19 therapeutics in a nationwide healthcare setting: a mixed-methods investigation
Source: Health Res Policy Syst. 2022 Dec 14;20:134. doi: 10.1186/s12961-022-00935-x (PMC9749626; doi:10.1186/s12961-022-00935-x)
Supplement: Supplementary file 1 — Additional file 1. COVID-19 treatment milestones. [file 12961_2022_935_MOESM1_ESM.pdf]

COVID-19 Treatment Milestones

* This form will record your name, please fill your name.

1. Person entering data

- ◯ WBE
- ◯ PM
- ◯ Other

2. Date of publication

Format: M/d/yyyy

3. Information source

- ◯ NIH treatment guidelines
- ◯ Pre-print
- ◯ Publication
- ◯ Other

4. Link to information source

5. Page where recommendation can be found

6. Medication Name

7. Recommendation type

- ◯ Use for all patients
- ◯ Use for patients with severe disease
- ◯ Use for ICU patients
- ◯ No recommendation for or against
- ◯ Do not use
- ◯ N/a- Not a guideline
- ◯ Other

8. Notes on patient population for use

9. Strength of recommendation

- ◯ A
- ◯ B
- ◯ C
- ◯ N/a - not a guideline
- ◯ Other

10. Quality of Evidence Underlying the recommendation

- ◯ I
- ◯ II
- ◯ III
- ◯ N/a- not a guideline
- ◯ Other

11. Notes

This content is neither created nor endorsed by Microsoft. The data you submit will be sent to the form owner.

Microsoft Forms
