# Supplementary material for: Factors associated with the speed and scope of diffusion of COVID-19 therapeutics in a nationwide healthcare setting: a mixed-methods investigation
Source: Health Res Policy Syst. 2022 Dec 14;20:134. doi: 10.1186/s12961-022-00935-x (PMC9749626; doi:10.1186/s12961-022-00935-x)
Supplement: Supplementary file 4 — Additional file 4. Table S1. Factors included in the diffusion matrix, their definitions, and impact of grading. [file 12961_2022_935_MOESM4_ESM.docx]

**Additional file 4: Table S1**. Factors included in the Diffusion Matrix, their Definitions, and Impact of Grading

| Factor | Definition | Grading/Impacts |
| --- | --- | --- |
| Biological and clinical plausibility | Theoretical, laboratory, and clinical basis for use of treatment/intervention. | High plausibility supports administration; low plausibility supports lack of use and rapid de-adoption |
| Conflicting evidence | Clinical evidence basis with similar or differing results. Higher quality clinical evidence given higher weight (e.g., clinical trials with more substantial impact than observational data). | Low conflicting evidence supports application of the treatment as found in the available evidence. High levels of conflicting evidence support lack of use and slower rates of uptake. |
| Strength of Recommendation in Clinical Guidelines | Per standard definitions (E.g., A is highest). | Higher levels of evidence (e.g. A), are associated with higher probability that the recommendation will be followed. Lower levels of evidence are associated with lower probability the recommendation will be incorporated into practice. |
| Level of evidence | Per standard definitions, the type of evidence supporting the recommendation. | I is the highest and most likely to lead to an intervention being adopted as recommended in the clinical guidelines. |
| Relative advantage* | The degree to which a new treatment has a clear and unambiguous advantage in either effectiveness or cost-effectiveness. | The higher the perceived benefit of the intervention, the more likely it is to be adopted. Interventions that are “first in-class” may have a particularly strong propensity to be adopted. |
| Compatibility with Clinical Needs* | The perception that an intervention is compatible with clinical needs, values, and norms. | The higher the perceived compatibility, the more likely it is to be adopted. |
| Observability* | The ability of the intervention to produce a change in clinical status that is measurable and observable for providers. | The more apparent a change in clinical status is, the stronger the impact of the effect. |
| Perceived risk of the intervention | The *perceived* risk of an intervention/potential to cause harm. | The higher the *perceived* risk of an intervention, the less likely it is to be adopted, and the more likely it is to be de-adopted. |
| Knowledge Required to Use the Intervention* | The amount of baseline knowledge a provider needs to use a clinical intervention. | The simpler the intervention, and the less knowledge that is required to administer it, the more likely it is to be adopted. |
| Familiarity with treatment | The degree to which clinicians are familiar with specific interventions in other contexts. | The more familiar and comfortable providers are with a treatment or a treatment class, the more likely that intervention is to be adopted. |
| Complexity of drug administration/monitoring* | The complexity of the intervention for the provider and the patient. This can include the complexity of drug administration or safety monitoring necessary. | Less complex and shorter-term interventions are more likely to be adopted than simpler and long-term interventions. |

*Adapted from Greenhalgh *et al*. Several variables included in Greenhalgh *et al* were not included in the Speed and Scope of Diffusion Matrix, as they were not considered to be relevant to short-term administration of medications for various reasons. Factors not graded include: trialability, re-invention, fuzzy boundaries, task issues, and augmentation/support.
