# Supplementary material for: Factors associated with the speed and scope of diffusion of COVID-19 therapeutics in a nationwide healthcare setting: a mixed-methods investigation
Source: Health Res Policy Syst. 2022 Dec 14;20:134. doi: 10.1186/s12961-022-00935-x (PMC9749626; doi:10.1186/s12961-022-00935-x)
Supplement: Supplementary file 5 — Additional file 5. Ivermectin prescribing. [file 12961_2022_935_MOESM5_ESM.pdf]

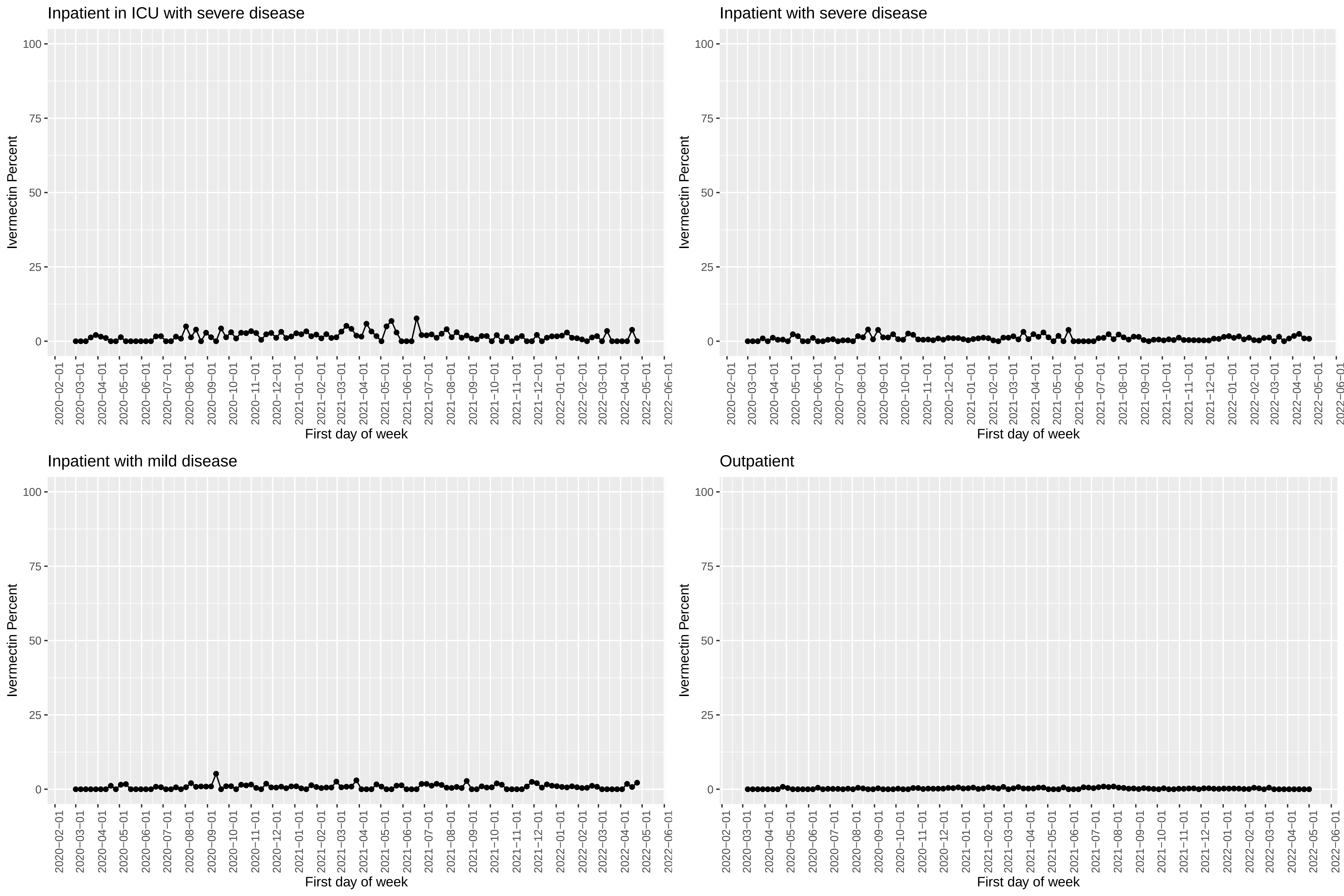
Inpatient in ICU with severe disease
Inpatient with severe disease
Inpatient with mild disease
Outpatient
Ivermectin Percent
100
75
50
25
0
First day of week
2020−02−01
2020−03−01
2020−04−01
2020−05−01
2020−06−01
2020−07−01
2020−08−01
2020−09−01
2020−10−01
2020−11−01
2020−12−01
2021−01−01
2021−02−01
2021−03−01
2021−04−01
2021−05−01
2021−06−01
2021−07−01
2021−08−01
2021−09−01
2021−10−01
2021−11−01
2021−12−01
2022−01−01
2022−02−01
2022−03−01
2022−04−01
2022−05−01
2022−06−01
